# Electroconvulsive therapy, electric field, neuroplasticity, and clinical outcomes

**DOI:** 10.1038/s41380-021-01380-y

**Published:** 2021-12-01

**Authors:** Zhi-De Deng, Miklos Argyelan, Jeremy Miller, Davin K. Quinn, Megan Lloyd, Thomas R. Jones, Joel Upston, Erik Erhardt, Shawn M. McClintock, Christopher C. Abbott

**Affiliations:** 1 Noninvasive Neuromodulation Unit, Experimental Therapeutics and Pathophysiology Branch, National Institute of Mental Health, National Institutes of Health, Bethesda, MD USA; 2 Department of Psychiatry and Behavioral Sciences, Duke University School of Medicine, Durham, NC USA; 3 Department of Psychiatry, The Zucker Hillside Hospital, Glen Oaks, NY USA; 4 Center for Neuroscience, Feinstein Institute for Medical Research, Manhasset, NY USA; 5 Zucker School of Medicine at Hofstra/Northwell, Department of Psychiatry, Hempstead, NY USA; 6 Department of Psychiatry, University of New Mexico, Albuquerque, NM USA; 7 Department of Mathematics and Statistics, University of New Mexico, Albuquerque, NM USA; 8 Division of Psychology, Department of Psychiatry, UT Southwestern Medical Center, Dallas, TX USA

**Keywords:** Depression, Prognostic markers

## Abstract

Electroconvulsive therapy (ECT) remains the gold-standard treatment for patients with depressive episodes, but the underlying mechanisms for antidepressant response and procedure-induced cognitive side effects have yet to be elucidated. Such mechanisms may be complex and involve certain ECT parameters and brain regions. Regarding parameters, the electrode placement (right unilateral or bitemporal) determines the geometric shape of the electric field (E-field), and amplitude determines the E-field magnitude in select brain regions (e.g., hippocampus). Here, we aim to determine the relationships between hippocampal E-field strength, hippocampal neuroplasticity, and antidepressant and cognitive outcomes. We used hippocampal E-fields and volumes generated from a randomized clinical trial that compared right unilateral electrode placement with different pulse amplitudes (600, 700, and 800 mA). Hippocampal E-field strength was variable but increased with each amplitude arm. We demonstrated a linear relationship between right hippocampal E-field and right hippocampal neuroplasticity. Right hippocampal neuroplasticity mediated right hippocampal E-field and antidepressant outcomes. In contrast, right hippocampal E-field was directly related to cognitive outcomes as measured by phonemic fluency. We used receiver operating characteristic curves to determine that the maximal right hippocampal E-field associated with cognitive safety was 112.5 V/m. Right hippocampal E-field strength was related to the whole-brain ratio of E-field strength per unit of stimulation current, but this whole-brain ratio was unrelated to antidepressant or cognitive outcomes. We discuss the implications of optimal hippocampal E-field dosing to maximize antidepressant outcomes and cognitive safety with individualized amplitudes.

## Introduction

Electroconvulsive therapy (ECT) remains the gold-standard treatment for patients with depressive episodes [1]. Independent of the antidepressant effect of ECT, many patients experience transient but debilitating cognitive side effects such as attention and memory impairment [2, 3]. ECT-mediated hippocampal neuroplasticity has been implicated in both antidepressant and cognitive outcomes [4, 5]. Neuroplasticity refers to the brain’s ability to restructure itself by forming new neural connections [6] and appears to be a common mechanism shared by both ECT and chemical antidepressant treatments [7]. Despite the possible relationship between hippocampal neuroplasticity and therapeutic mechanisms (neurogenesis, synaptogenesis, gliogenesis, angiogenesis), the relationship between hippocampal neuroplasticity and antidepressant response has been inconclusive. Some investigations demonstrated a direct relationship between hippocampal neuroplasticity and antidepressant response [8–13], but other investigations were negative [14–21]. The rapid hippocampal volume increase involves extensive remodeling and may also be related to procedure-related cognitive impairment [22, 23]. Consistent with this mechanism, recent ECT-imaging investigations have demonstrated a direct relationship between hippocampal neuroplasticity and impaired cognitive performance [5, 24].

Right unilateral electrode placement predominately stimulates the right hemisphere in an effort to reduce cognitive side effects [3]. The right hemisphere stimulation results in greater right hippocampal neuroplasticity that disentangles the effect from the generalized seizure [8, 10, 13, 21, 25–27]. Recent work on electric field (E-field) modeling has explored the relationship between electric field strength and neuroplasticity. Concerning ECT parameters, the electrode placement determines the geometric shape and the amplitude determines the E-field magnitude within this geometric shape [28]. Spherical head models have demonstrated that the E-field is influenced by individual anatomic variability (skull thickness, head diameter, and brain volume) [29]. The application of E-field modeling to ECT investigations has demonstrated a direct relationship between E-field and neuroplasticity, [30] but the relationship between E-field and antidepressant outcome has been mixed [30, 31]. To date, there has been no published examination of the E-field strength and longitudinal cognitive performance during an ECT series.

In standard ECT clinical practice, pulse amplitude tends to be fixed at 800 or 900 milliamperes (mA) without clinical or scientific rationale [28]. Lower pulse amplitudes reduce the magnitude of the induced E-field which could potentially decrease the risk of cognitive side effects. Case reports and recent clinical trials have demonstrated that 500–600 mA is sufficient to generate seizure activity with fewer cognitive side effects [32–35]. We recently completed a clinical trial of adults and older adults with depression who were randomized to right unilateral electrode placement ECT treatment with different pulse amplitude (600, 700, and 800 mA) with a bitemporal (800 mA) contingency [36]. The results of this investigation demonstrated a trade-off between cognitive safety (improved with 600 mA arm) and antidepressant response (improved with 700 and 800 mA arms). The cognitive measure most associated with amplitude mediated cognitive impairment was the Delis Kaplan Executive Function System (DKEFS) Verbal Fluency Test, which assesses frontal-temporal cognitive functions (e.g., verbal fluency, cognitive flexibility).

Based on the variability from fixed extracranial amplitude to E-field strength, we shift the focus from extracranial amplitude to hippocampal E-field strength. Here, we aim to determine the relationships between hippocampal E-field strength, hippocampal neuroplasticity, and antidepressant and cognitive outcomes. We hypothesize that E-field mediated hippocampal neuroplasticity will be associated with both antidepressant and cognitive outcomes as measured by the Hamilton Depression Rating Scale-24 item (HDRS_24_) and DKEFS Verbal Fluency Test, respectively. Recognizing that hippocampal neuroplasticity may be related to both antidepressant and cognitive outcomes, we further hypothesize that an optimal ECT E-field may result in sufficient hippocampal neuroplasticity for antidepressant response with cognitive safety.

## Methods

### Participants, assessments, ECT, and study design

The overall study design and clinical outcomes have been previously described (ClinicalTrials.gov Identifier: NCT02999269) [36]. The University of New Mexico Human Research Protections Office approved this investigation. All subjects provided written informed consent to the research protocol and study participation. Subjects had a diagnosis of major depressive disorder (MDD; single episode or recurrent, severe with or without psychotic features), met clinical indication for ECT, were right-handed, and the age ranged between 50 and 80 years. Scheduled medications were tapered before the initiation of ECT, but as-needed medications including quetiapine (maximal cumulative dose per day: 200 mg), trazodone (200 mg), and lorazepam (3 mg) were permitted.

**Fig. 1:**
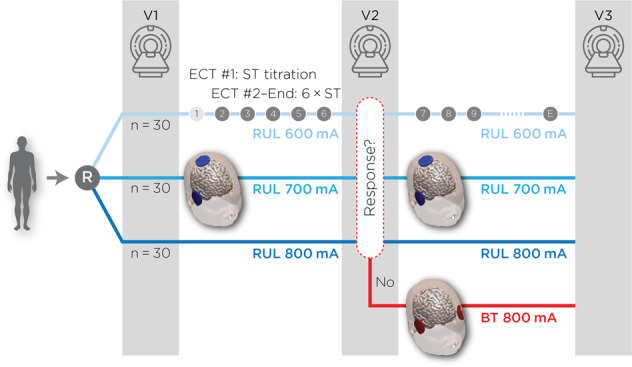
Overall study design. After randomization to either 600, 700, or 800 mA amplitudes, subjects received antidepressant ratings, neuropsychological assessments, and imaging pre-ECT (Visit 1 or “V1”), mid-ECT (V2), and post-ECT (V3). If subjects failed to respond to their assigned amplitude at the V2 assessment (defined as <25% reduction in pre-ECT HDRS_24_), subjects completed the protocol with bitemporal electrode placement.

All subjects started the ECT series with right unilateral (RUL) electrode placement [37] and were randomized to 600, 700, and 800 mA treatment arms. Subjects commenced ECT with ultrabrief (0.3 milliseconds (ms)) pulse width until a planned interim data analysis. Because of a trend of reduced antidepressant outcomes in the 600 mA arm, subjects (*n* = 15) enrolled in the latter portion of this investigation received brief (1.0 ms) pulse width. The rationale of the change in pulse width from 0.3 to 1.0 ms was to improve the antidepressant outcomes in the lower amplitude arms based on the strength-duration curve (lower amplitudes may require longer pulse width). The first ECT session determined individual seizure thresholds with subsequent treatments provided at six times the seizure threshold with similar adjustments to pulse train duration and frequency across all amplitude arms [38]. Subjects received clinical, cognitive, and imaging assessments pre- (V1), mid- (after the sixth ECT treatment, V2), and post-ECT (within one week of finishing the ECT series, V3). The Hamilton Depression Rating Scale-24 item (HDRS_24_) was the primary antidepressant outcome measure [39]. If subjects were non-responsive to the assigned pulse amplitude (<25% reduction in from baseline HDRS_24_ at the second visit, *n* = 20), subjects then received bitemporal (BT) electrode placement (800 mA, 1.0 ms pulse width) for the remainder of the ECT series (Fig. 1) [40]. The cognitive battery included a comprehensive assessment of multiple cognitive domains. Here, we focus on the results from the DKEFS Verbal Fluency Test, the cognitive test most associated with amplitude mediated cognitive impairment measure that assessed phonemic fluency, semantic fluency, and cognitive flexibility [36, 41].

### MRI acquisition and pre-processing

3T-Siemens scanner acquired T1 data with the following parameters: Repetition time (TR) = 2530 milliseconds (ms), echo time (TE) = 1.64, 3.5, 5.36, 7.22, 9.08 ms, Inversion time (TI) = 1200 ms, flip angle = 7.0°, slices = 192, field of view = 256, matrix 256 × 256, voxel size = 1.0 × 1.0 × 1.0 millimeter (mm) and total acquisition time 6:03 (minutes:seconds). T2 data was collected with the following parameters: TR = 2530 ms, TE = 474 ms, flip angle = 120.0°, slices = 192, field of view = 256, matrix 256 × 256, voxel size = 1.0 × 1.0 × 1.0 mm and total acquisition time = 5:09. FreeSurfer 6.0 segmented the cortical and subcortical anatomy with a longitudinal pipeline [42, 43]. This provides a robust and reliable estimation of the subcortical volumes and cortical thickness by creating an unbiased within-subject template image using inverse consistent registration [44, 45]. We processed all the time points separately with the default FreeSurfer workflow and created an unbiased template from all the time points for each subject. Once this template was created, parcellations and segmentation were carried out at each time point initialized with common information from the within-subject template [43]. We identified the bilateral hippocampal volumes and calculated the percent changes in these regions relative to the pre-treatment volume.

### E-field modeling

We used the Simulation of Non-Invasive Brain Stimulation (SimNIBS) software for E-field modeling [46]. SimNIBS creates a subject-specific, anatomically realistic volume conductor model. The T1- and T2-weighted scans are segmented into skin, bone, eyes, cerebral spinal fluid, ventricles, and gray and white matter with a combination of FMRIB Software Library (FSL) [47] and Statistical Parametric Mapping 12 (SPM12) Computational Anatomy Toolbox [48, 49]. SimNIBS then turns this segmentation into a tetrahedral head mesh using Gmsh, a three-dimensional finite element (FE) mesh generator. Gmsh provide unique conductivity values for each tissue type: cerebrospinal fluid: (1.654 Siemens/meter (S/m)), vitreous bodies (0.50 S/m), scalp (0.465 S/m), gray matter (0.275 S/m), white matter (0.126 S/m), spongy bone (0.025 S/m), and compact bone (0.0008 S/m) [46]. ECT electrodes are added to the head mesh in either RUL or BT configuration and stimulated with the corresponding current. SimNIBS then uses a FE solver to calculate the voltages and electric fields that correspond to the stimulation throughout the head mesh.

We calculated the right and left hippocampal E-field strength based on the electrode placement (RUL or BT) and amplitude (600, 700, 800 mA) from the last treatment of the ECT series. Hippocampal E-field strength (*E*_*hippo*_) was calculated as the 95th percentile of E-field magnitudes from all voxels in the hippocampus, serving as an estimate of the peak-induced field strength while avoiding the influence of tissue boundary effects that could bias the absolute maximum E-field values. To balance the focused approach on the hippocampus, we also calculated *E*_*brain*_*/I*_*electrode*_, where *E*_*brain*_ is the 90th percentile of E-field magnitude in the whole brain and *I*_*elecrode*_ is the stimulation current [50]. This ratio is the induced E-field in the brain per unit of stimulation current. This metric depends only on the electrode placement and individual head anatomy and is independent of waveform parameters including current amplitude, and thus reflects only the spatial properties of the induced E-field in the brain.

### Statistical analyses

#### Amplitude and hippocampal E-field

With RUL electrode placement and the initially assigned amplitude (600, 700, or 800 mA), we assessed hippocampal E-field variability across amplitudes in the right and left hippocampi with a one-way analysis of variance and follow-up contrasts to determine amplitude differences with *E*_*hippo*_.

#### Hippocampal E-field and volume change

We assessed the relationship between right hippocampal E-field strength (*E*_*r-hippo*_) and right hippocampal volume change (percent change relative to pre-ECT volume), (∆Vol_*r-hippo*_*/Pre-ECT-Vol*_*r-hipp*_) with a linear regression analysis controlling for sex, age, pulse width and number of treatments. We calculated effect sizes (partial eta squared) for dependent variables of interest for all linear models.

#### Hippocampal E-field, volume change, and clinical outcomes

We assessed the relationships with *E*_*r-hippo*_, right hippocampal volume change, and antidepressant outcomes (percent change in HDRS relative to pre-ECT HDRS or %∆HDRS) with similar linear models controlling for sex, age, pulse width and treatment number. In parallel, we assessed the relationships with *E*_*r-hippo*_, right hippocampal volume change, and cognitive outcomes (change in DKEFS Letter Fluency or ∆DKEFS Letter Fluency) controlling for sex, pulse width, treatment number, and Test of Premorbid Functioning (TOPF) to control for premorbid intelligence [51]. Electrode placement influences hippocampal E-field strength through E-field geometry and was not included as a covariate. Age was accounted for in the DKEFS Letter and Category Fluency demographic-adjusted scaled score and therefore not included as a covariate in the cognitive analyses. Using structural equation modeling, we assessed the mediation effect of hippocampal volume change (∆Vol_*r-hippo*_) on the association between *E*_*r-hippo*_ and %∆HDRS. We quantified the direct effect of *E*_*r-hippo*_ on %∆HDRS (coefficient c’), the indirect effect mediated by ∆Vol_*r-hippo*_ (coefficient a*b), as well as the total effect (coefficient c), controlling for age and sex. Likewise, we assessed the mediation of the effect of ∆Vol_*r-hippo*_ on the association between *E*_*r-hippo*_ and ∆DKEFS-LF (Fig. 4F). Model fits were assessed by the root mean square error of approximation (RMSEA), the comparative fit index (CFI), and the Tucker-Lewis index (TLI). RMSEA smaller than 0.06 and a CFI and TLI larger than 0.95 indicate relatively good model fit [52]. Finally, we performed receiver operating characteristic (ROC) analysis, using *E*_*r-hippo*_ as a classifier of negative cognitive outcome. We determined the area under the ROC curve with the binary classifier of the lower bound of test-retest reliability of DKEFS Letter Fluency Scaled Score (−3) [41]. The 95% confidence interval for the area under the curve (AUC) is computed with 2000 stratified bootstrap replicates. Finally, we performed similar analysis with a control brain region, the right postcentral gyrus, which receives higher E-field compared to the hippocampus but is thought to be unrelated to clinical outcome.

#### Hippocampal E-field, E_brain_/I, and clinical outcomes

*E*_*brain*_*/I*_*electrode*_ is a whole brain E-fied metric independent of stimulation parameters other than electrode placement. Contrasting *E*_*brain*_*/I*_*electrode*_ with the hippocampal results will assess the anatomic specificity of the clinical outcomes. We assessed the relationship between *E*_*r-hippo*_ and *E*_*brain*_*/I* controlling for age and sex. We assessed the relationships between *E*_*brain*_*/I* and antidepressant outcomes controlling for sex, age, and number of treatments. We then assessed the relationship between *E*_*brain*_*/I* and cognitive outcome controlling for TOPF, sex, and number of treatments.

## Results

The demographic and clinical characteristics of the study sample are summarized in Supplementary Material (Supplement Material Table 1). Because of the mid-series electrode placement switch and the large difference in the left hippocampal E-field between the RUL and BT electrode placements, we focused on the right hippocampus. Because of the effect of time on hippocampal volume change (Supplementary Fig. 1), we restricted the analysis to subjects who completed the study protocol (*n* = 52). We present the results on subjects who completed the protocol with RUL electrode placement, DKEFS Category Fluency, left hippocampus and right postcentral gyrus (control region) in Supplementary Material (Supplementary Material, Sections 1–4).

### Amplitude and Hippocampal E-field

**Fig. 2:**
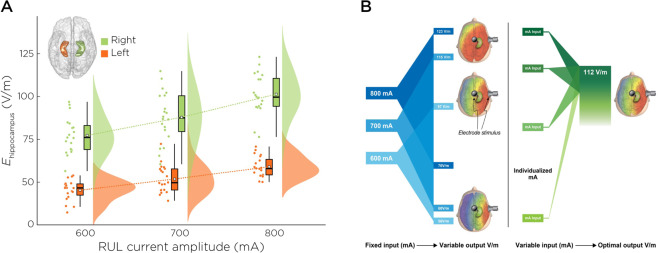
Hippocampal E-field variability and implications for ECT dosing. **A** Right and left hippocampal E-field strengths across the three amplitude arms. **B** Individualized amplitudes have the potential to create accurate and precise E-field dosing.

The average (± standard deviation) *E*_*r-hippo*_ increased across the 600, 700, and 800 mA amplitude arms: 77.5 Volts/meter (V/m) (± 11.3), 87.0 V/m (±14.8), and 101.0 V/m (±12.3) (F_2,50_ = 14.2, *p* < 0.01). The contrasts between the 600/700 mA arms have similar *E*_*r-hippo*_ (difference: 9.5 V/m, *p* = 0.11 with Bonferroni correction). The contrasts between the 600/800 mA (difference: 23.5 V/m, *p* < 0.01) and 700/800 mA arms (difference: 14.0 V/m, *p* = 0.006) reflect amplitude differences with *E*_*r-hippo*_ (Fig. 2A, translational implications in Fig. 2B).

### Hippocampal E-field and volume change

**Fig. 3:**
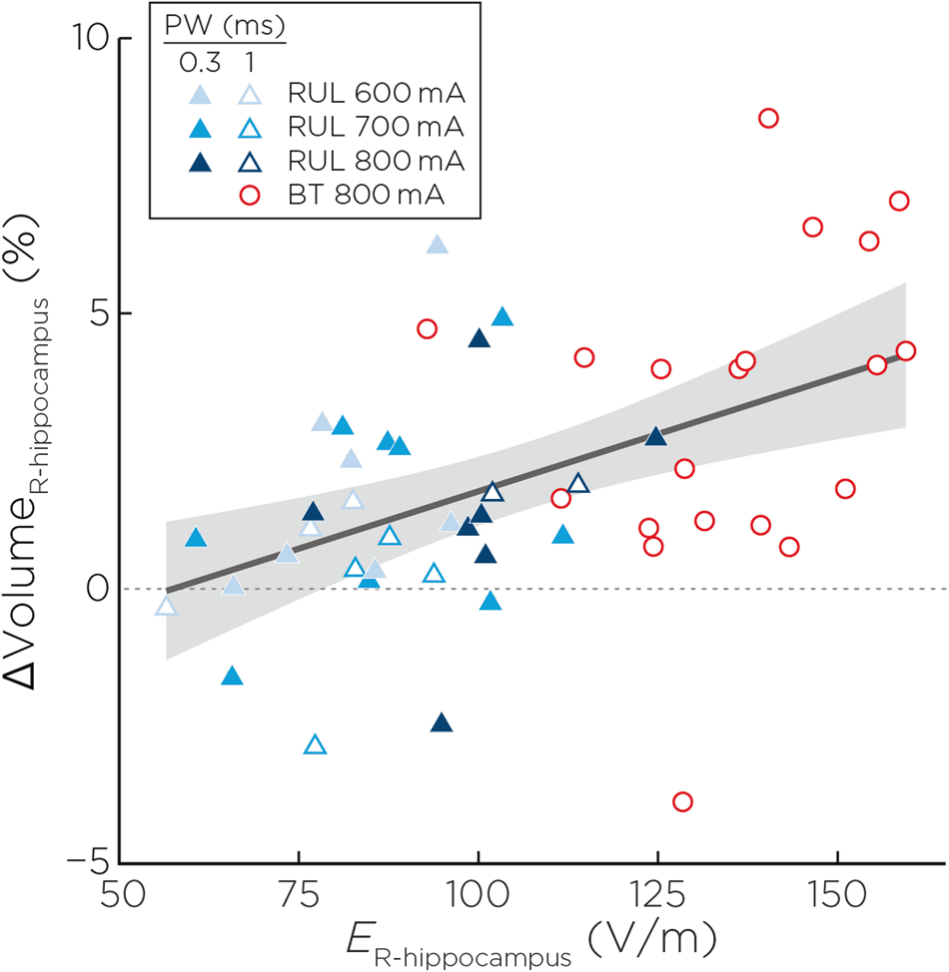
E-field and right hippocampal neuroplasticity. Right hippocampal E-field was associated with right hippocampal neuroplasticity (t_46_ = 2.59, *p* = 0.01) controlling for age, sex, pulse width and number of treatments.

*E*_*r-hippo*_ had a direct relationship with right hippocampal volume change (*R*^2^ = 0.34, β = 0.04, t_47_ = 2.52, *p* = 0.02, partial eta [2] effect size = 0.13) (Fig. 3). Sex was also associated (β = 1.43, t_46_ = 2.59, *p* = 0.01). Post-estimation sex contrasts revealed that females had 1.43% greater hippocampal volume change relative to males (standard error ± 0.68, confidence interval: 0.06–2.79).

### Hippocampal E-field, volume change, and clinical outcomes

**Fig. 4:**
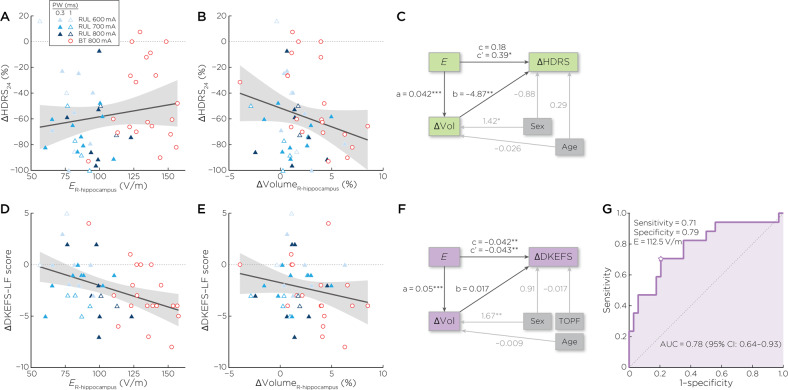
Hippocampal neuroplasticity and clinical outcomes. **A** Right hippocampal E-field was not related to antidepressant outcomes (t_46_ = −0.62, *p* = 0.54). **B** Right hippocampal neuroplasticity had a relationship with antidepressant outcomes (t_46_ = −3.12, *p* = 0.003). **C** Structural equation modeling showed that right hippocampal neuroplasticity mediated the relationship between right hippocampal E-field strength and antidepressant response. **D** Right hippocampal E-field was associated with change in DKEFS Letter Fluency (t_44_ = −2.44, *p* = 0.02). **E** Right hippocampal neuroplasticity was not related to change in DKEFS Letter Fluency (t_44_ = −0.68, *p* = 0.50). **F** Structural equation modeling showed that right hippocampal neuroplasticity did not mediate the direct effect of right hippocampal E-field and change in DKEFS Letter Fluency. **G** Receiver operator characteristic curve analysis comparing right hippocampal E-field and cognitive outcome (change in DKEFS Letter Fluency > −3) revealed an area under the curve of 0.78 (95% CI: 0.64–0.93) and maximal sensitivity and specificity of 112.5 V/m.

Hippocampal E-field, Hippocampal Volume Change, and Antidepressant Outcomes: *E*_*r-hippo*_ had no relationship with %∆HDRS (*R*^2^ = 0.15, β = −0.13, t_46_ = −0.62, *p* = 0.54, effect size = 0.008). Right hippocampal volume change had a relationship with %∆HDRS (*R*^2^ = 0.26, β = −5.45, t_46_ = −3.12, *p* = 0.003, effect size = 0.17). ECT treatment number also contributed to %∆HDRS (β = 4.11, t_46_ = 3.00, *p* = 0.004, effect size = 0.16). Structural equation modeling showed that there was a significant direct effect of *E*_*r-hippo*_ on %∆HDRS (c’ = 0.39; z = 2.43, *p* = 0.015) and a significant indirect effect through ∆Vol_*r-hippo*_ (a = 0.042, *b* = −4.87; z = −2.21, *p* < 0.027), leading to an insignificant total effect (c = c’ + a × b = 0.18; z = 1.24, *p* = 0.22) (Fig. 4A–C). The RMSEA for the model is < 0.001; the CFI and TLI for the model are 1.000 and 1.046, respectively, altogether indicating a good model fit.

Hippocampal E-field, Hippocampal Volume Change, and Cognitive Outcomes: *E*_*r-hippo*_ had an inverse relationship with ∆DKEFS Letter Fluency (*R*^2^ = 0.24, β = −0.04, t_44_ = −2.44, *p* = 0.02, effect size = 0.12). Right hippocampal volume change had no relationship with ∆DKEFS Letter Fluency (*R*^2^ = 0.15, β = −0.14, t_44_ = −0.68, *p* = 0.50, effect size = 0.01). Structural equation modeling showed a significant direct effect of *E*_*r-hippo*_ on ∆DKEFS Letter Fluency (c’ = −0.043; z = −2.61, *p* = 0.009) and significant total effect (c = −0.042; z = −3.16, *p* = 0.002) independent of changes in right hippocampal volume change (indirect effect: z = 0.09, *p* = 0.93). The RMSEA for the model is <0.001; the CFI and TLI for the model are both 1.00, altogether indicating a good model fit. Receiver operating characteristic curve for *E*_*r-hippo*_ and ∆DKEFS Letter Fluency revealed an area under the curve of 0.78 (95% CI: 0.64–0.93) and maximal sensitivity and specificity of 112.5 V/m (Fig. 4D–G).

### Hippocampal E-field, E_brain_/I_electrode_, and clinical outcomes

**Fig. 5:**
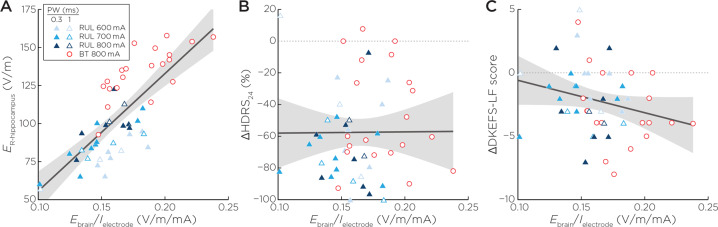
Right hippocampal E-field (*E*_*r-hippo*_), ratio of the induced E-field in the brain per unit of stimulation current (*E*_*brain*_*/I*_*electrode*_), antidepressant, and cognition outcomes. **A** *E*_*r-hippo*_ had a direct relationship with *E*_*brain*_*/I*_*electrode*_ (t_48_ = 8.19, *p* < 0.001). **B** *E*_*brain*_*/I*_*electrode*_ was not associated with %∆HDRS (t_47_ = −0.40, *p* = 0.69). **C** *E*_*brain*_*/I*_*electrode*_ was not associated with ∆DKEFS Letter Fluency (t_45_ = −1.66, *p* = 0.10).

*E*_*r-hippo*_ had a direct relationship with *E*_*brain*_*/I*_*electrode*_ (*R*^2^ = 0.61, β = 0.0008, t_48_ = 8.19, *p* < 0.001, effect size = 0.61). Sex was also associated with *E*_*brain*_*/I*_*electrode*_ (β = 0.01, t_49_ = 2.11, *p* = 0.04, effect size = 0.08). Post-estimation sex contrasts revealed that females had 0.01 greater *E*_*brain*_*/I*_*electrode*_ relative to males (standard error ± 0.006, confidence interval: 0.0005–0.02). *E*_*brain*_*/I*_*electrode*_ was unassociated with %∆HDRS (*R*^2^ = 0.18, β  = −65.20, t_47_ = −0.40, *p* = 0.70, effect size = 0.003) or ∆DKEFS Letter Fluency (*R*^2^ = 0.18, β = −24.46, t_45_ = −1.66, *p* = 0.10, effect size = 0.06) (Fig. 5A–C).

## Discussion

This investigation provided new insights focused on the relationships between hippocampal E-field strength, neuroplasticity, and clinical and cognitive outcomes. First, the average right hippocampal E-field strength increased with each amplitude arm. However, despite the incremental amplitude difference of 100 mA across the arms, the induced E-fields within each arm exhibit substantial variability, which is large enough to mask group differences (i.e., no E-field differences between 600 and 700-mA arms). Second, right hippocampal E-field strength is associated with hippocampal neuroplasticity, as measured by increase in hippocampal volume after ECT. Third, right hippocampal neuroplasticity mediated the relationship between right hippocampal E-field strength and antidepressant outcome. Fourth, right hippocampal E-field strength had a direct relationship with the cognitive outcome (specifically phonemic fluency). Unlike antidepressant outcomes, right hippocampal neuroplasticity did not mediate this relationship between right hippocampal E-field and cognitive outcomes. Receiver operating characteristic curves identified 112.5 V/m as the maximal right hippocampal E-field associated with cognitive safety. Fifth, the whole brain metric (*E*_*brain*_*/I*) did not demonstrate the same relationships as the right hippocompal E-field suggesting anatomic specificity for these relationships.

Recent investigations have examined the relationships between E-field strength, neuroplasticity, and antidepressant outcomes. The Global ECT MRI Collaboration (GEMRIC) database, which included 151 subjects treated with RUL at 800 or 900 mA, revealed a direct relationship with left hippocampal E-field strength and left hippocampal neuroplasticity [30]. Our results demonstrated the direct relationship between E-field strength and neuroplasticity in both the right and left hippocampi (left hippocampal results presented in Supplementary Material Section 3). The higher amplitudes (800 and 900 mA) used in the GEMRIC database may have created a ceiling effect with the right hippocampal neuroplasticity that was not evident with the lower amplitudes (600, 700, and 800 mA) used in the present investigation. Despite the robust sample size, the GEMRIC investigation failed to establish a relationship between E-field strength and antidepressant outcome. In contrast, a recent investigation that included mixed electrode placements (both RUL and BT) at 900 mA showed that higher E-field strength in both the left and right temporal lobes was associated with worse antidepressant outcome [31]. Our investigation demonstrated that hippocampal neuroplasticity is a potential mediator in the E-field and antidepressant relationship such that increased E-field strength is associated with increased hippocampal neuroplasticity, which is associated with improved antidepressant outcomes.

While hippocampal E-field dose is related to both hippocampal neuroplasticity and cognitive outcomes, the link between hippocampal neuroplasticity and cognitive impairment is less robust. Our original cognitive hypothesis focused on hippocampal memory function as measured with the Hopkins Verbal Learning Test-Revised (HVLT-R) Percent Retention Raw Score [53], but verbal memory retention was not related to amplitude-mediated differences in cognitive performance [36]. The pathophysiology of ECT-mediated cognitive impairment may not be limited to hippocampal neuroplasticity, and thus alternative anatomic locations (e.g., prefrontal cortex) and mechanisms such as disrupted long-term potentiation and related impact on resting-state functional connectivity should be considered [54, 55]. Even if the hippocampus is not directly involved in verbal fluency performance, the hippocampus appears to be the most sensitive anatomic region for measuring the impact of E-field dosing on cognition for the rest of the brain. In contrast to antidepressant outcomes (higher E-field is better), cognitive outcomes suggest the opposite relationship (higher E-field compromises cognitive safety). Our results are demonstrated an optimal E-field strength 112.5 V/m in the right hippocampus to maximize antidepressant outcomes without compromising cognitive safety.

Several limitations should be considered when interpreting these results. First, seizure is an important therapeutic component of ECT [56], but the impact of seizure on neuroplasticity and clinical outcomes was not assessed with this investigation. Seizure activity may be related to neuroplasticity with and without E-field generation (i.e., Metrazol therapy) [57, 58]. The lateralization of ictal power with RUL electrode placement suggests that E-field and ictal power may be interrelated [59]. Future work may employ similar structural equation models with topographical ictal power to disentangle the impact of E-field and ictal power on ECT’s therapeutic and iatrogenic effects. Second, our investigation included subjects who switched to BT electrode placement and 1.0 ms pulse width. BT results in a different E-field spatial distribution compared to RUL with higher E-field strength in the hippocampus. We repeated our analysis with RUL electrode placement only and maintained our relationship with E-field and cognitive outcomes (Supplementary Material Section 1). Future work will incorporate the impact of E-field as a vector field to better elucidate the impact of both E-field strength and direction (BT right-left E-field direction, RUL anterior-posterior) related to differences in clinical and cognitive outcomes related to electrode placement switch [60]. Third, the E-field modeling used T1 and T2 structural scans. Earlier E-field modeling approaches used diffusion tensor imaging (DTI), and demonstrated that in a single head model, the relative error of the E-field magnitude in the isotropic versus anisotropic head model within the right hippocampus approaches 15% [61]. However, a validation study using in vivo intracranial recordings in humans demonstrated that white matter anisotropy did not significantly improve E-field modeling accuracy [62]. In recent work by Takamiya et al., E-field models were computed in thirty depressed subjects receiving RUL ECT, a subgroup of which incorporated DTI-derived anisotropic conductivity in the models. The E-field correlations with regional brain volumetric changes were similar between both models [57]. Future work will need to validate E-field modeling accuracy (with or without DTI) prior to clinical use (“E-field-informed ECT”). Fourth, our sample included a relatively homogenous sample of older adults with MDD meeting the indication to start with RUL electrode placement. Our sample did not include subjects that required initiation of the ECT series with BT electrode placement secondary to acuity and need for rapid response. Larger samples across the adult lifespan and with different acuity levels will be needed to further assess these complex relationships [63].

## Conclusions and future directions

Our results have implications for ECT dosing. With a fixed extracranial current amplitude, the ECT “dose” as represented by the intracranial E-field is highly variable due to anatomic differences in skin, skull, fluid, and brain tissue [29]. This anatomic variability is prominent in older (age 50+ years) adults with MDD and can compromise both antidepressant efficacy (insufficient stimulation of mood-related circuitry) and safety (inducing cognitive impairment due to excessive stimulation of cognitive related circuitry). The E-field variability precludes the use of a fixed amplitude for older adults with MDD treated with ECT. Our results suggest a sweet spot of ECT dosing ~ 112.5 V/m in the right hippocampus that will maintain antidepressant benefit and maximize cognitive safety.

To improve the accuracy and precision of ECT dosing, we propose a solution towards individualized amplitude (Fig. 2B). “E-field-informed-ECT” has the potential to individualize current amplitude based on the knowledge of the location (hippocampus) and strength (112.5 V/m) of the target region [64]. This method would require pre-ECT imaging (MRI T1, T2) and the capacity to perform E-field modeling before starting ECT. While achieving the goal of individualized amplitude and reducing variability of the ECT dose, the time, equipment, and expertise required for this option could have limited translational impact. Alternatively, amplitude titrated seizure thresholds will reduce the individual variability related to fixed amplitude ECT dosing [50, 65]. Amplitude titrated seizure threshold will result in consistent E-field strength despite variance in anatomic features typically associated with E-field variability, and potentially limit over-exposure of the brain to excessive stimulation [66]. Reducing the E-field variability will create a more standardized and consistent ECT dosing strategy for treatment with RUL ECT, thus improving antidepressant and cognitive outcomes.

## Supplementary information

Supplemental Material
