# Supplementary material for: Electroconvulsive therapy, electric field, neuroplasticity, and clinical outcomes
Source: Mol Psychiatry. 2021 Dec 1;27(3):1676–82. doi: 10.1038/s41380-021-01380-y (PMC9095458; doi:10.1038/s41380-021-01380-y)
Supplement: Supplementary file 1 — Supplemental Material [file 41380_2021_1380_MOESM1_ESM.docx]

Supplemental Material

**Supplemental Table 1: Clinical and demographic characteristics of the study sample**

| Clinical and demographic features | 600mA (n = 15) mean (SD) | 700mA (n = 19) mean (SD) | 800mA (n = 18) mean (SD) | F or χ^2^ (p-value) |
| --- | --- | --- | --- | --- |
| Age (years) | 65.5 (8.7) | 64.4 (6.8) | 66.9 (10.8) | 0.36 (0.70) |
| Sex: Male/Female | 3/12 | 4/15 | 8/10 | 3.3 (0.20) |
| Single depressive episode/recurrent | 1/14 | 4/15 | 1/17 | 2.67 (0.26) |
| Psychotic/non-psychotic | 5/10 | 7/12 | 6/12 | 0.07 (1.0) |
| Depressive episode duration (months) | 14.9 (15.7) | 14.4 (21.5) | 25.6 (28.9) | 1.32 (0.28) |
| Number of depressive episodes | 5.3 (5.5) | 2.9 (3.0) | 3.6 (2.6) | 1.57 (0.22) |
| Age of onset (years) | 38.3 (19.0) | 39.8 (22.8) | 32.4 (18.0) | 0.68 (0.51) |
| Lifetime duration (years) | 5.0 (4.2) | 7.0 (12.7) | 9.4 (11.8) | 0.72 (0.49) |
| Framingham Stroke Risk Profile (raw score) | 8.3 (3.9) | 7.9 (4.4) | 8.9 (3.9) | 0.25 (0.78) |
| ECT Appropriateness Scale | 7.6 (1.8) | 8.4 (1.6) | 8.3 (1.4) | 1.11 (0.34) |
| Maudsley Treatment Failure | 2.1 (1.1) | 1.8 (1.0) | 2.2 (1.2) | 0.50 (0.61) |
| Treatment and Antidepressant outcomes |  |  |  |  |
| Pre-ECT HDRS-24 | 37.9 (8.2) | 37.2 (6.4) | 33.2 (6.8) | 2.00 (0.15) |
| Post-ECT HDRS-24 | 23.7 (10.6) | 13.6 (9.8) | 15.5 (8.3) | 4.68 (0.01) |
| Treatment number | 10.5 (2.8) | 11.0 (3.5) | 11.5 (3.2) | 0.42 (0.66) |
| Last treatment: RUL/BT* | 10/5 | 13/6 | 9/9 | 1.56 (0.46) |
| %∆ HDRS-24 | 48.3 (33.1) | 66.5 (27.6) | 55.7 (29.1) | 1.62 (0.21) |
| Cognitive outcomes |  |  |  |  |
| Baseline MoCA total score | 22.6 (3.2) | 23.9 (3.3) | 24.9 (3.3) | 1.89 (0.16) |
| Test of Premorbid Function | 105.0 (13.5) | 111.1 (12.7) | 109.4 (9.4) | 1.10 (0.34) |
| ∆ DKEFS Verbal Fluency - Category Fluency Scaled Score | -1.9 (3.8) | -2.2 (3.0) | -1.9 (5.4) | 0.03 (0.97) |
| ∆ DKEFS Verbal Fluency - Letter Fluency Scaled Score | -1.3 (3.1) | -2.8 (1.9) | -2.4 (3.0) | 1.37 (0.26) |

|  |
| --- |
| HDRS-24 = Hamilton Depression Rating Scale - 24 items  RUL = right unilateral electrode placement, BT = bitemporal electrode placement |
| DKEFS = Delis Kaplan Executive Function System  MoCA = Montreal Cognitive Assessment |

**Supplemental Figure 1: Right hippocampal longitudinal change by visit**

**
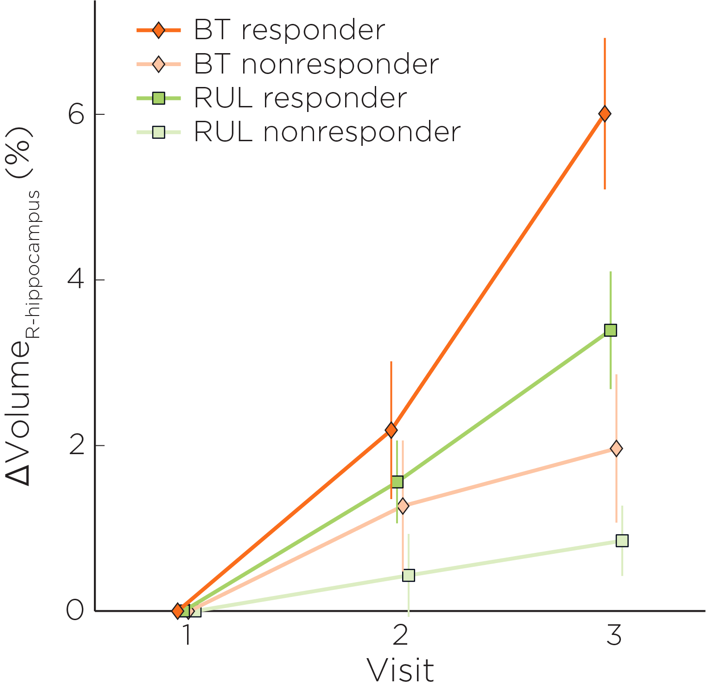
**

S Figure 1. Legend. Because of the effect of time on hippocampal Volume Change, we restricted the analysis to subjects that completed the study protocol with a third post-ECT visit (V3) (n = 52). In the above figure, the percent change in right hippocampal volume is on the y-axis and the study visit is on the x-axis. All subjects started with right unilateral (RUL) electrode placement (V1). If a subject was RUL non-responsive at the second visit (< 25% change in HDRS_24_ relative to baseline), the subject then received bitemporal (BT) electrode placement for the remainder of the ECT series. Orange colors reflect subjects that received a BT switch and green colors reflect subjects that completed the series with RUL.

**Supplemental Material Section 1: Right unilateral electrode placement results**

Thirty-two subjects completed the protocol with RUL electrode placement.

*Hippocampal E-field and Volume Change*

*E_r-hippo_* did not have a relationship with right hippocampal volume change (R^2^ = 0.36, $\beta$ = 0.03, t_26_ = 1.74, p = 0.09, effect size = 0.10). Sex also contributed to the model ($\beta$ = 1.71, t_26_ = 2.39, p = 0.02, effect size = 0.18).

*Hippocampal E-field, Volume Change and Clinical Outcomes*

Hippocampal E-field, Hippocampal Volume Change and Antidepressant Outcomes: *E_r-hippo_* did not have a relationship with %∆HDRS (R^2^ = 0.21, $\beta$ = -0.51, t_26_ = −1.59, p = 0.12, effect size = 0.09). Right hippocampal volume change did not have a relationship with %∆HDRS (R^2^ = 0.23, $\beta$ = -5.36, t_26_ = −1.84, p = 0.08, effect size = 0.11).

Hippocampal E-field, Hippocampal Volume Change and Cognitive Outcomes: *E_r-hippo_* had a relationship with ∆DKEFS Letter Fluency (R^2^ = 0.19, $\beta$ = -0.06, t_26_ = −2.08, p = 0.047, effect size = 0.14). Right hippocampal volume change did not have a relationship with ∆DKEFS Letter Fluency (R^2^ = 0.07, $\beta$ = 0.13, t_26_ = −0.43, p = 0.67, effect size = 0.01).

**Supplemental Material Section 2: DKEFS Category Fluency results**

The main manuscript included results from DKEFS Letter Fluency results. The following results are for DKEFS Category Fluency from RUL and BT subjects who completed the study protocol.

*Hippocampal E-field, Volume Change and Clinical Outcomes*

Hippocampal E-field, Hippocampal Volume Change and Cognitive Outcomes: *E_r-hippo_* did not have a relationship with ∆DKEFS Category Fluency (R^2^ = 0.14, $\beta$ = -0.03, t_43_ = -1.13, p = 0.26, effect size = 0.03). Right hippocampal volume change did not have a relationship with ∆DKEFS Category Fluency (R^2^ = 0.12, $\beta$ = -0.18, t_43_ = −0.55, p = 0.58, effect size = 0.01).

**Supplemental Material Section 3: Left hippocampus results**

*Amplitude and Hippocampal E-field*

The average (+/− standard deviation) *E_l-hippo_* increased across the 600, 700, and 800 mA amplitude arms: 46.0 Volts/meter (V/m) (+/− 5.9), 51.4 V/m (+/− 8.2), and 58.1 V/m (+/− 5.5) (F_2,50_ = 13.94, p < 0.01). The contrasts between the 600/700 mA arms have similar *E_l-hippo_* (difference: 5.5 V/m, p = 0.06 with Bonferroni correction). The contrasts between the 600/800 mA (difference: 12.1 V/m, p < 0.01) and 700/800 mA arms (difference: 6.7 V/m, p = 0.01) reflect amplitude differences with *E_l-hippo_*.

*Hippocampal E-field and Volume Change*

*E_l-hippo_* did not have a direct relationship with left hippocampal volume change (R^2^ = 0.40, $\beta$ = 0.02, t_48_ = 1.42, p = 0.16, effect size = 0.04). Age ($\beta$ = -0,10, t_46_ = −2.49, p = 0.02, effect size = 0.12) and sex ($\beta$ = 1.83, t_46_ = 2.35, p = 0.02, effect size = 0.11) contributed to the model.

*Hippocampal E-field, Volume Change and Clinical Outcomes*

Hippocampal E-field, Hippocampal Volume Change and Antidepressant Outcomes: *E_l-hippo_* did not have a relationship with %∆HDRS (R^2^ = 0,16, $\beta$ = -0.18, t_46_ = −1.03, p = 0.31, effect size = 0.02). Left hippocampal volume change did have a relationship with %∆HDRS (R^2^ = 0.24, $\beta$ = -3.80, t_46_ = −2.34, p = 0.02, effect size = 0.11). ECT treatment number ($\beta$ = 3.68, t_46_ = 2.60, p = 0.01, effect size = 0.13) also contributed to the model.

Hippocampal E-field, Hippocampal Volume Change and Cognitive Outcomes: *E_l-hippo_* did not have a relationship with ∆DKEFS Letter Fluency (R^2^ = 0.16, $\beta$ = -0.02, t_44_ = −1.07, p = 0.29, effect size = 0.03). Left hippocampal volume change did not have a relationship with ∆DKEFS Letter Fluency (R^2^ = 0.16, $\beta$ = -0.18, t_44_ = −1.13, p = 0.26, effect size = 0.03).

**Supplemental Material Section 4: Right postcentral gyrus (control region)**

*Right postcentral gyrus E-field and Volume Change*

*E_r-postcentral_* did not have a direct relationship with right postcentral gyrus volume change (R^2^ = 0.09, $\beta$ = 0.005, t_47_ = 0.20, p = 0.84, effect size = 0.0009).

*Right postcentral gyrus E-field, Volume Change and Clinical Outcomes*

Hippocampal E-field, Hippocampal Volume Change and Antidepressant Outcomes: *E_r-postcentral_* did not have a relationship with %∆HDRS (R^2^ = 0.16, $\beta$ = -0.16, t_47_ = −1.00, p = 0.32, effect size = 0.02). Right postcentral gyrus volume change did not have a relationship with %∆HDRS (R^2^ = 0.14, $\beta$ = -0.26, t_46_ = −0.27, p = 0.79, effect size = 0.002).

Hippocampal E-field, Hippocampal Volume Change and Cognitive Outcomes: *E_r-postcentral_* did not have a relationship with ∆DKEFS Letter Fluency (R^2^ = 0.17, $\beta$ = -0.02, t_45_ = −1.30, p = 0.20, effect size = 0.04). Right postcentral gyrus volume change did not have a relationship with ∆DKEFS Letter Fluency (R^2^ = 0.15, $\beta$ = -0.08, t_45_ = −0.94, p = 0.35, effect size = 0.02).
